# Inverse design in photonic crystals

**DOI:** 10.1515/nanoph-2023-0750

**Published:** 2024-02-05

**Authors:** Ruhuan Deng, Wenzhe Liu, Lei Shi

**Affiliations:** State Key Laboratory of Surface Physics, Key Laboratory of Micro- and Nano-Photonic Structures (Ministry of Education), and Department of Physics, Fudan University, Shanghai 200433, China; State Key Laboratory of Surface Physics, Institute for Nanoelectronic Devices and Quantum Computing, Fudan University, Shanghai, 200438, China

**Keywords:** inverse design, photonic crystals, nanophotonics, optimization

## Abstract

Photonic crystals are periodic dielectric structures that possess a wealth of physical characteristics. Owing to the unique way they interact with the light, they provide new degrees of freedom to precisely modulate the electromagnetic fields, and have received extensive research in both academia and industry. At the same time, fueled by the advances in computer science, inverse design strategies are gradually being used to efficiently produce on-demand devices in various domains. As a result, the interdisciplinary area combining photonic crystals and inverse design emerges and flourishes. Here, we review the recent progress for the application of inverse design in photonic crystals. We start with a brief introduction of the background, then mainly discuss the optimizations of various physical properties of photonic crystals, from eigenproperties to response-based properties, and end up with an outlook for the future directions. Throughout the paper, we emphasize some insightful works and their design algorithms, and aim to give a guidance for readers in this emerging field.

## 1 Introduction

**Figure 1:**
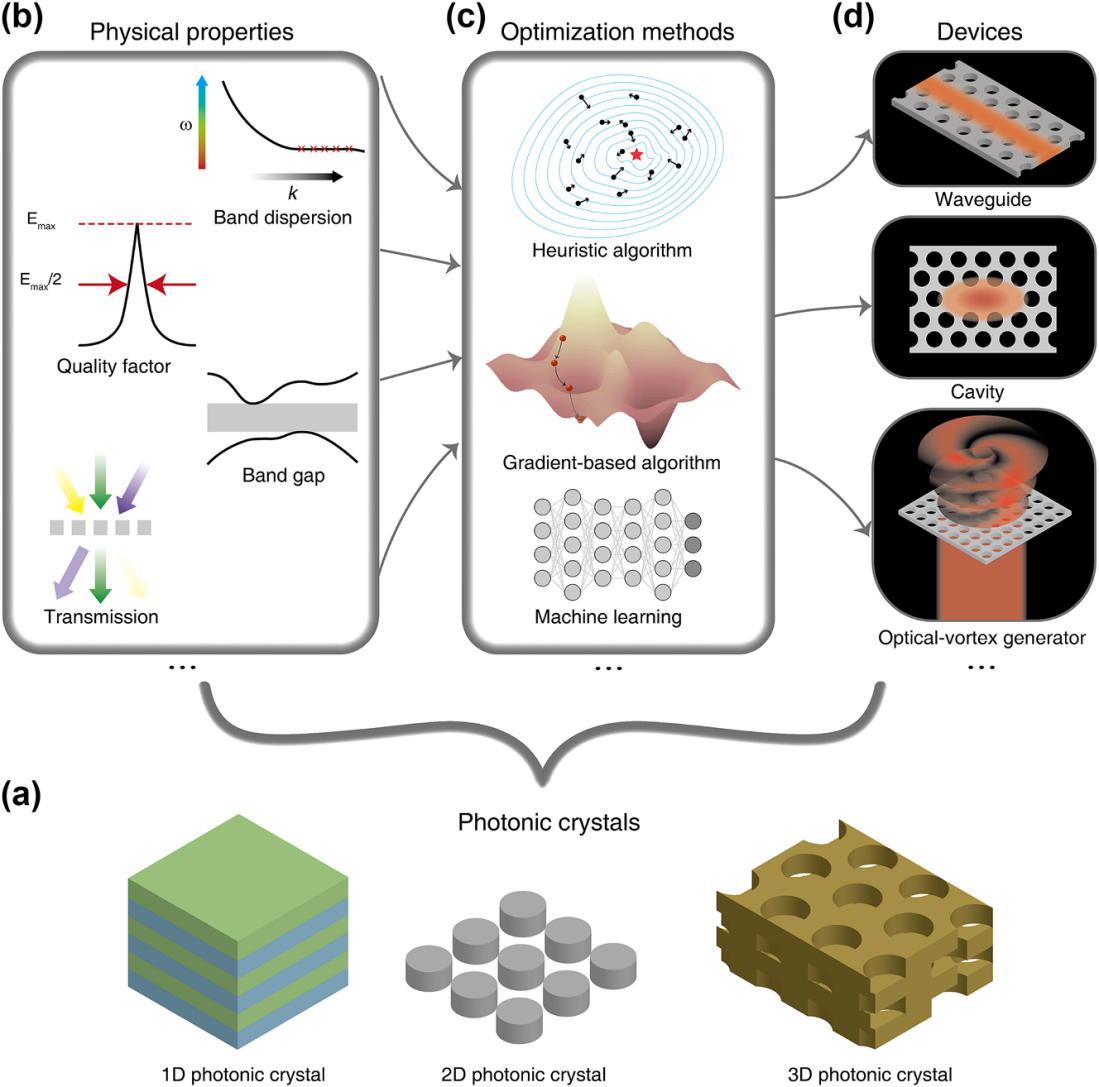
Diagram on inverse design of photonic crystals. (a) Different types of photonic crystals (1D, 2D and 3D photonic crystals). (b) Different physical properties of photonic crystals (band gap, band dispersion, quality factor, transmission and so on). (c) Different optimization methods to design photonic crystals (Heuriatic algorithm, gradient-based algorithm, machine learning, and so on). (d) Different devices can be designed by optimizing their physical properties (waveguide, cavity, optical-vortex generator, and so on).

Inspired by the periodic arrangement of atoms or molecules in crystals, Yablonovitch [1] and John [2] proposed artificial dielectric structures with periodicity known as photonic crystals (see Figure 1(a)). As the periodicity in crystals brings about numerous fascinating physical phenomena related to band structures in condensed matter physics, photonic crystals’ periodic structures also lead to a wealth of photonic band-related properties [3], [4] (see Figure 1(b)), and thus they are able to manipulate light in an unprecedented and more effective way. To date, photonic crystals have realized a number of interesting physical phenomena like complete band gaps [5], slow light [6], and photon localization [7], and also have a wide range of applications in many fields, such as optical communication [8], sensor [9], and imaging [10].

In order to design a photonic crystal that meets requirements, intuition-based approaches are frequently used [11], [12]. During these traditional optimization processes, the overall structure is inspired by the priori known physical effect, and the trial and error further determines its specific parameters that make the deigned photonic crystal work most effectively. While this kind of method has performed well in the past, it faces increasing challenges now as the design objectives become more complex. This is due to the fact that the geometry is always confined to regular structures with a small number of tuning parameters for constraints of time and computational resources. Recently, with the rapid development in computer science, a new design strategy called inverse design has been proposed and is evolving quickly [13]. Leveraging effective algorithms, inverse design methods can automatically optimize the structure of the photonic crystal to improve its figure-of-merit (FoM), a user-defined metric that can characterize the device’s performance, and achieve the design target. And benefiting from the rich parameter freedom opened by the optimization algorithm, inverse design can produce high performance structures, often with irregular and inspiring shapes.

Algorithms used in inverse design can be roughly classified into three categories: Heuristic algorithms, gradient-based algorithms, and deep learning algorithms (see Figure 1(c)). A heuristic algorithm is usually the one that is informed by a natural phenomenon. For example, particle swarm algorithms [14] mimic the behavior of birds’ predation by scattering many data points in a parameter space, and assigning each data point a speed to evolve under certain rules based on its FoM, as shown in the upper panel in Figure 1(c). This type of algorithm is widely used in inverse design for their ease of implementation, and it better explores the parameter space compared to the parameter sweeping method. However, as the number of design parameter increased, its computationally expansive nature still makes its application to inverse design difficult and even sometimes infeasible, and this renders gradient-based algorithms stand out. A gradient-based algorithm uses the information of gradients [15], the derivative of the FoM with respect to all the design parameters, to update the parameters in every iteration (see the middle panel in Figure 1(c)), thus every step it takes is more judicious and efficient, and the optimization can converge more quickly. While the calculation of gradients itself needs considerable computational resources, the adjoint method [16] is proposed so that only two simulations are needed to obtain gradients regardless of the number of design parameters, which eventually makes gradient-based inverse design practical. Because the number of design parameters no longer limits the optimization, topology optimization [17], which was originally developed in mechanical engineering, is always applied to fully broaden the design space, where every pixel or voxel of the structure is a design parameter. To focus our review, we will not detail the implementation and discussion on adjoint method and topology optimization, instead we refer interested readers to the papers related to two topics [18], [19], [20], [21]. Very recently, breakthroughs in deep learning have revolutionized many domains, such as computer vision [22], natural language processing [23], and decision making [24], and it also paves a new avenue for inverse design. Deep learning algorithms are achieved by neural networks [25], which use the construction of multilayered ‘neuron’ to realize the functionality of prediction, classification and generation (see the lower panel in Figure 1(c)). Different types of deep neural networks (DNNs), including convolutional neural networks [26] (CNNs), recurrent neural networks [27] (RNNs), and generative adversarial networks [28] (GANs), are used in inverse design of photonic crystals, and are promising to produce accurate and fast design. While both heuristic and gradient-based algorithms need to start the optimization from scratch for different inverse design problems, deep learning algorithms are potential to provide a once-for-all solution, because of its generalization ability. More content about this topic can be found in the relevant reviews [29], [30], [31]. It is worth mentioning that deep learning has also been widely used to inverse design other metamaterials to optimize their various properties, such as transmission [32], [33], reflection [34], [35], [36], [37], absorption [38], and so on. For example, Liu’s group applied a hybrid neural network and a VAE-based generative model to predict and inverse design reflective metasurfaces. They discussed the interdependence of optical responses at different frequencies, and pushed the limits of metasurfaces’ multifunctional capabilities [37]. Notice that there are also other types of algorithms besides these three categories, like some semidefinite programming (SDP)-based methods [39], which are used to solve semidefinite programming, a kind of convex optimization that is suitable for various problems including inverse design. They do not directly calculate the gradient, but can iterate using other mathematical techniques.

During the past few decades, inverse design methods utilizing above algorithms have been used to produce various photonic crystal-based devices. Notice that although these devices have different functionalities, such as wave guiding, light trapping, and vortex beam generation (see Figure 1(d)), and have distinct application scenarios varying from optical analog computing to image processing, their optimizations can always be concentrated on the specific physical properties of the photonic crystal. Thus, in this review we focus on the physical properties being optimized to discuss the recent progress in the field of inverse design for photonic crystals. More specifically, in Section 2 we summarize the works about inverse design for its eigenproperties, including band gap, band dispersion, quality factor, local density of states, and topological properties. Section 3 surveys the works on inverse design of response-based properties such as transmission, for both on-chip devices and free-space devices, and more complicated ones that are constructed from the excited electromagnetic fields. Finally, Section 4 summarizes this paper and prospects for the future directions of this emerging field.

## 2 Inverse design for the photonic crystals’ eigenproperties

### 2.1 Inverse design for band gap

Band gap is one of the most important characteristics of photonic crystals, since a target of designing photonic crystals is to achieve complete band gaps that suppress the spontaneous emission and prohibit the light propagation in the structure [1], [2]. Thus, optimizing photonic band gap is a popular topic in inverse design. In 1999, Cox and Dobson [42] maximized band gaps in two-dimensional photonic crystals for E-polarization modes using a gradient-based algorithm. The problem is formulated as

$$\underset{\rho \in ad}{\sup}\underset{\alpha \in K}{\inf}\min\left\{\lambda_{j+1}\left(\rho ,\alpha \right)-\omega_{0}^{2},\omega_{0}^{2}-\lambda_{j}\left(\rho ,\alpha \right)\right\}$$

where *ρ* is structure parameters in the admissible set ad, *α* is the bloch wave vector in the first Brillouin zone *K*, *λ*_*j*_ is the *j*th nonnegative eigenvalue, and *ω*_0_ is the designate frequency position of the band gap. While the gradient of the fundamental element in the FoM, i.e. ∂_*ρ*_ *λ*_*j*_(*ρ*, *α*), can be calculated directly, the minimization operation and the degenerate eigenvalue problem hinder the calculation of the classical gradient with respect to the FoM. This problem is solved by introducing the generalized gradient that can be defined on non-smooth functions [15], and the geometry is restricted to be symmetric to reduce the number of considered *α*. This method can significantly enlarge the band gap from an initial guess with a small gap, and the case of H-polarization modes is considered later in a similar fashion [43]. Afterwards, Kao et al. [44] applied level set methods with the same projected generalized gradient ascent algorithm, and obtained completely discrete two-dimensional square lattices that exhibit the largest band gaps at that time. Here, the level set method is a way to represent the geometry which can ensure its binary structure [45]. However, the direct dependence of the generalized gradient makes the method often suffer from numerical difficulties and convergence problems because of the lack of regularity. A new approach was proposed to reformulate the problem as a convex semidefinite optimization problem that can be effectively solved by SDP-based methods [46]. Thus, it circumvented the direct calculation of generalized gradients and made the formulation more regularized. Also, the FoM was set to be a more physically meaningful value, the gap-midgap ratio, and the high-dimensional subspaces used in the optimization were shrunk to make the problem small-scale and solvable.

**Figure 2:**
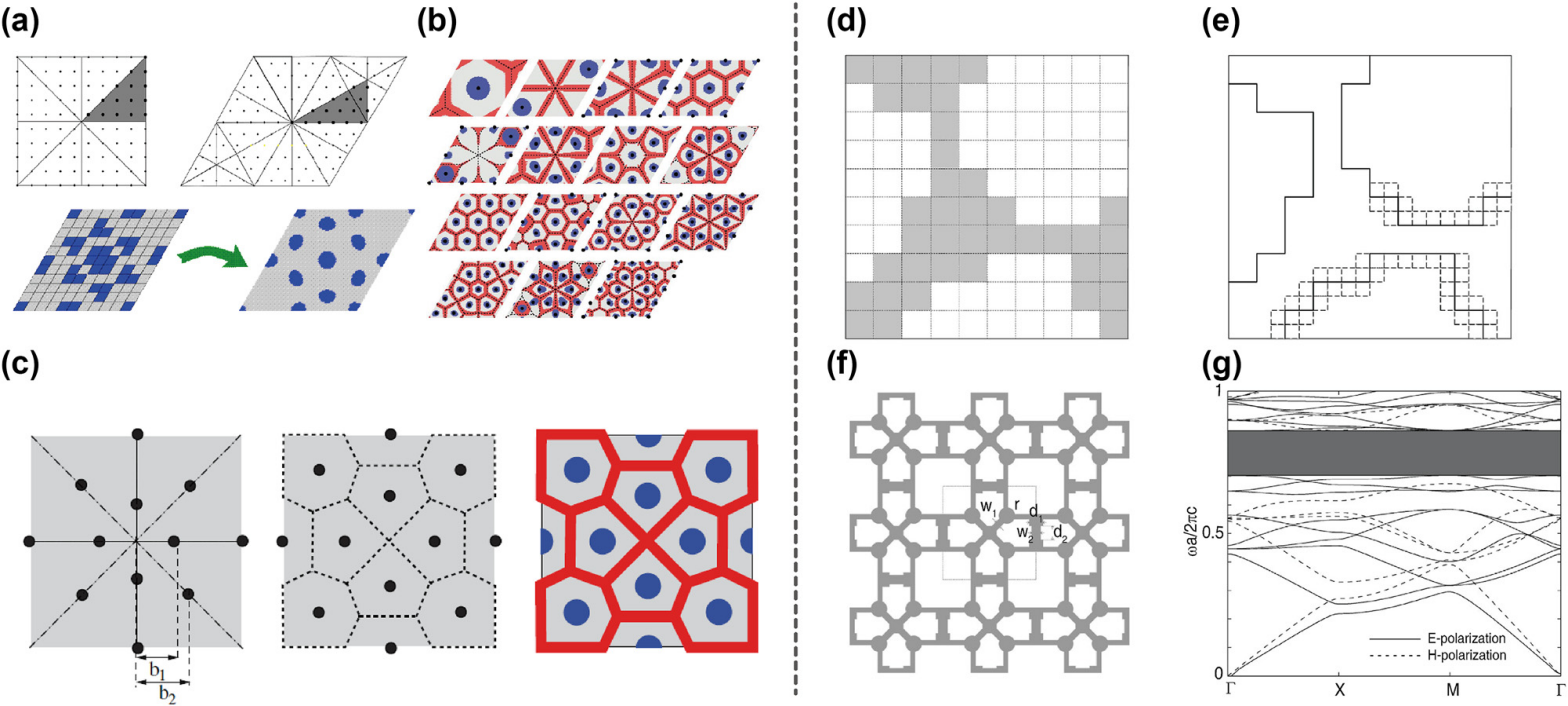
Inverse design for band gap. (a) Schematic of the two-step optimization procedure. (b) The optimized structures for rhombic unit cell from *n* = 1 to *n* = 15. Red is the distribution for TE polarization, and blue is for the TM polarization, while black dots and dashed lines indicate the structures constructed from the observed rules. (c) The construction process for optimal structures on a square cell using Lloyd’s algorithm and Voronoi tessellations. (d) The optimized structure on coarse grids. (e) The refined structure with the encoding area around edges with dashed squares. (f) The simplified structure. (g) The band structure of the optimized photonic crystal. (a)–(c) Are adapted with permission from Ref. [40], and (d)–(g) are adapted with permission from Ref. [41].

As algorithms for optimizing photonic band gaps have been introduced, many efforts were made to use these algorithms to achieve more complicated objectives. Sigmund and Hougaard [40] used gradient-based topology optimization to investigate optimal structures for gaps between bands *n* and *n* + 1. Because of the large number of design variables, this work took a two-step optimization process, where a thorough search on coarse grids was done first to provide candidates for the next procedure. Then candidates were mapped to finer grids, and they were set as initial guesses for the topology optimization, where gradients were used to find final optimal fine grid structures, as shown in Figure 2(a). After systematic optimizations for band gaps from *n* = 1 to *n* = 15, different structures that maximized specific band gaps were generated (see Figure 2(b) for rhombic unit cells), and closer observation led to the conclusion that optimal structures for gaps between band *n* and *n* + 1 can be obtained by constructing centroidal Voronoi tessellations with *n* points [47]. More specifically, optimal TM structures were made by finding the minimum energy distribution of *n* points using Lloyd’s algorithm, and walls of the corresponding Voronoi tessellations made up optimal TE structures, as shown in Figure 2(c). This result provides understanding and support for previous findings about planar band gap structures [44], also it indicates one direction for the application of inverse design in photonic crystals, which is discovering the physical laws and exploring the limits. Later, Men et al. [48] utilized an SDP-based algorithm with adaptive computational mesh to design photonic crystals with multiple band gaps, complete band gaps, and even multiple complete band gaps. They also discussed the trade-off between different band gaps and the existence of numerous local optima. It was found that the structures supporting multiple band gaps are always complex and hard to get from traditional design methods. This demonstrates the importance of inverse design for increasingly complicated goals. The optimization of band gaps was further incorporated into the designing process to inverse design photonic topological insulators (TIs) that is beyond traditional models [49], and techniques mentioned above were used to create multifunctional photonic TIs that can, for example, support dual-polarization [50] or multiband corner states [51]. For the development in this field, readers can refer to a recent review [52].

In addition to gradient-based algorithms, Heuristic and deep learning algorithms have also been used to optimize band gaps [41], [53], [54]. Shen et al. [41] designed 2D photonic crystals with largest absolute band gap using genetic algorithm. During the optimization, fill patterns of pixels in unit cells were encoded in ‘chromosomes’ with binary digits, corresponding to two dielectric materials. Then in every generation, populations were generated by selectively mating and mutating current ‘chromosomes’ to find the global optimum after iterations. In order to reduce the computational time and make the structures practical for fabrication, a two-stage strategy was introduced. In the first stage, coarse-grid patterns were used to encode ‘chromosomes’ as shown in Figure 2(d). In the second stage, patterns were refined and only those around structures generated in the first stage were used to encode the ‘chromosomes’ (see Figure 2(e)). As the result converges, the obtained structure suggested a simple structure with smooth circular columns, which is consistent with previously discovered laws that E-polarization band gaps favored isolated disk patterns and H-polarization band gaps favored connected walls patterns (see Figure 2(f)). The final structure was given by further optimizing the geometric parameters and reached a relative absolute band gap of 20.1 % (see Figure 2(g)).

Above discussions are all for two dimensional (2D) photonic crystals, while the inverse design of 3D photonic crystals was also considered. Men et al. [55] applied the SDP formulation to design 3D photonic crystals with complete band gaps for the first time. The symmetric structure was obtained by performing all symmetry operations on the smallest asymmetric unit parallelepiped, and a robust topology optimization was used to include fabrication uncertainties. Although only a little improvement was made compared with hand designs, it discovered many new structures to be examined. Nussbaum et al. [56] presented a 3D method to optimize the photonic crystal slab (PCS), where the semi-analytical approach called guided mode expansion is used to calculate eigenvalues at bands. To get the gradient, an automatic differentiation technique from computer science was utilized, which will be discussed in details later in other section. During the optimization, the position and size of the holes in a PCS are allowed to vary, and finally generated a waveguide with significantly improved bandwidth.

### 2.2 Inverse design for band dispersion

Band gap is an important characteristic of the band structure, whereas band dispersion is another more comprehensive one. Fortunately, optimizations of band dispersion are very similar to optimizations of band gap as building blocks of the FoM are all eigenvalues at bands. While the optimization of a generic band dispersion has been done for demonstration using gradient-based algorithms [59], most dispersion-engineering problems focus on flat band with low group-velocity dispersion (GVD) [57], [60], [61], [62], [63], which is useful for many devices, thus we will start with and mainly discuss this area, but notice that these methods can be easily utilized to optimize arbitrary dispersion relations.

**Figure 3:**
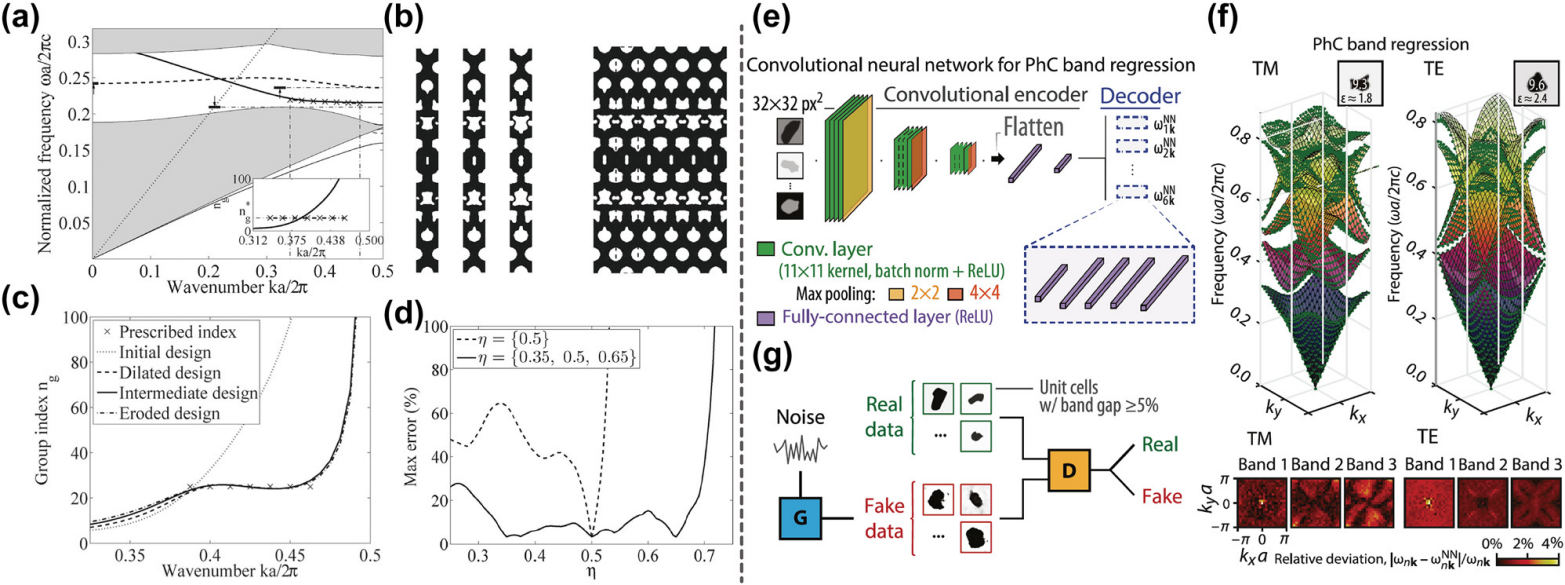
Inverse design for band dispersion. (a) Schematic of the objective and constraints in the robust formulation. (b) The designed structures. Left: the dilated, intermediate, and eroded designs. Right: the PhCW constructed from the intermediate design. (c) The group index of different designs and the prescribed one. (d) The maximum error between the actual group index and the target group index for different *η* values under normal and robust optimization. (e) The architecture of the band-prediction neural network. (f) Prediction results of the neural network. Top: examples of the predicted band structures for both TM and TE polarization. Bottom: the relative error between the predicted and actual frequencies for different bands. (g) Schematic of the generative adversarial network. (a)–(d) Are adapted with permission from Ref. [57], and (e)–(g) are adapted with permission from Ref. [58].

In 2011, Wang et al. [57] applied robust topology optimization to design slow light photonic crystal waveguides (PhCWs). In order to tailor the dispersion properties, the FoM was set as differences between desired group indexes with current ones calculated from direct differentiation to eigenfrequencies, and band constraints were used to keep upper and lower bands away from the designed one, so that the multimode interference can be avoided (see Figure 3(a)). This work also introduced a projection method to achieve robust topology optimization, where the dilated, intermediate, and eroded designs of supercells were considered simultaneously to take the manufacturing error into account, and constructing PhCWs accordingly (see Figure 3(b)). A design result is shown in Figure 3(c). It can be seen that band dispersions were well in line with the prescribed one for both the perfect and the perturbed structures. Figure 3(d) better illustrates the advantage of the robust formulation compared with the normal formulation. *η* is a tuning parameter that determines the variation of geometries, and *η* equals 0.35, 0.5, and 0.65, corresponding to dilated, intermediate and eroded structures respectively. It is shown that robust optimization ensures the optimized structure preserves good performance under fabrication imperfections, and this is important for subsequent practical applications. The paper went on to optimize PhCWs with smaller GVD, broader bandwidth, and even different constant group indexes, revealing power and versatile of inverse design methods. Afterwards, a wavelength-tunable, large-angle steering optical phased array (OPA) was design with a similar method [64]. First the band dispersion was optimized to generate slow-light PhCWs by setting the FoM as pairwise frequency differences for a number of parallel wavevectors. Then the radiation was introduced by adding periodic perturbations. To make the originally designed dispersion unchanged, the inverse design algorithm was again used to increase the imaginary part of eigenfrequencies for the radiation loss and decrease the deviation from the previous dispersion relation at the same time. Mode couplers were also produced using inverse design to make a complete photonic crystal circuit.

Besides algorithms concerning the calculation of eigenfrequencies and their corresponding gradients, many other inspiring methods were also proposed. Stainko and Sigmund [63] modeled the problem by setting harmonically varying even and odd excitations, and evaluated the dispersion with self-defined variables measuring mode confinements. Because of its use with excitation fields, the adjoint method was applied to calculate the gradient, and gradient-based topology optimization was used later. The measurement of confinements enables the algorithm to automatically find guided modes whose fields are concentrated in the center region, and an artificial damping term was included to avoid sharp resonance peaks that maybe troublesome for the optimization. This method successfully produced a PhCW with a constant group velocity and another one with designated dispersion. Wang et al. [60] circumvented finite difference approximation by analytically evaluated group velocities and directly set them as design targets. Under this formulation, the gradient can also be calculated using the adjoint method. Furthermore, this work made a detailed analysis for PhCW optimizations. It discussed ways to confine modes and reduce field losses, and discovered trade-off between average group velocities and modal confinements under different group velocity errors. This quantitatively describes the negative correlation between two quantities to a certain degree.

Many non-gradient based algorithms have also been utilized to predict and design band dispersions for photonic crystals [54], [58], [65], [66]. Christensen et al. [58] constructed convolutional neural networks for the prediction of the first six TE or TM bands. The neural network consists of an encoder and a decoder as shown in Figure 3(e), where the encoder takes discretized unit cell patterns of structures as the input and compresses them into lower-dimensional abstract representations through three convolutional layers and two fully-connected layers, and the decoder contains six feed-forward networks, each predicting their corresponding band dispersions from abstract representations through five fully-connected layers. In Figure 3(f), test results show good agreements between network predictions (green markers) and reference calculations (surfaces), and the low relative deviation between predictions and ground truths is better represented in the lower panel for different bands. While the forward neural network can be incorporated into the tandem network to inverse design band dispersions avoiding the one-to-many problem [54], this work chose GANs for the inverse design of bandgaps as shown in Figure 3(g). The generator takes noises in and produces candidate structures, and the discriminator decides whether the structure satisfies requirements. Different variants of GAN were tested, and they all ‘learned’ the recipe to produce desired band gaps, indicating their abilities to inverse design band dispersions. Recently, a new method based on Fourier synthesis was proposed to inverse design the dispersion of photonic-crystal resonators (PhCRs) [65]. In this approach, the optimization of dispersions for multiple mode numbers was decomposed into individual ones for single mode number, and each individual optimization was solved easily without gradients, then final structures were generated by Fourier synthesis. This idea of decoupling FoMs resembles the design for metasurfaces, and has the potential to improve the capability and flexibility of inverse design for photonic crystals.

### 2.3 Inverse design for quality factors and local density of states (LDOS)

The quality (*Q*) factor is a very essential property that describes the decay rate of the energy [3]. A higher *Q* factor means photons can be confined in a longer time therefore enhancing the interaction between light and matter. Thus, photonic crystals that possess eigenmodes with high *Q* factors are widely used in many applications such as sensing [9], lasing [69] and nonlinear frequency-conversions [70]. Since the *Q* factor can be written as the ratio between the real and imaginary part of eigenfrequencies, it can be optimized using gradient-based algorithms with the help of the automatic differentiation [59]. However, as the optimization landscape of devices with high-*Q* resonances is always complex and nonconvex [71], the optimization against them easily gets trapped in local minima, meanwhile although heuristic algorithms can address this problem to some extent, it is at the cost of a longer evaluation time. Therefore, neural networks are often used to speed up the optimization [67], [68], [72], [73], [74].

**Figure 4:**
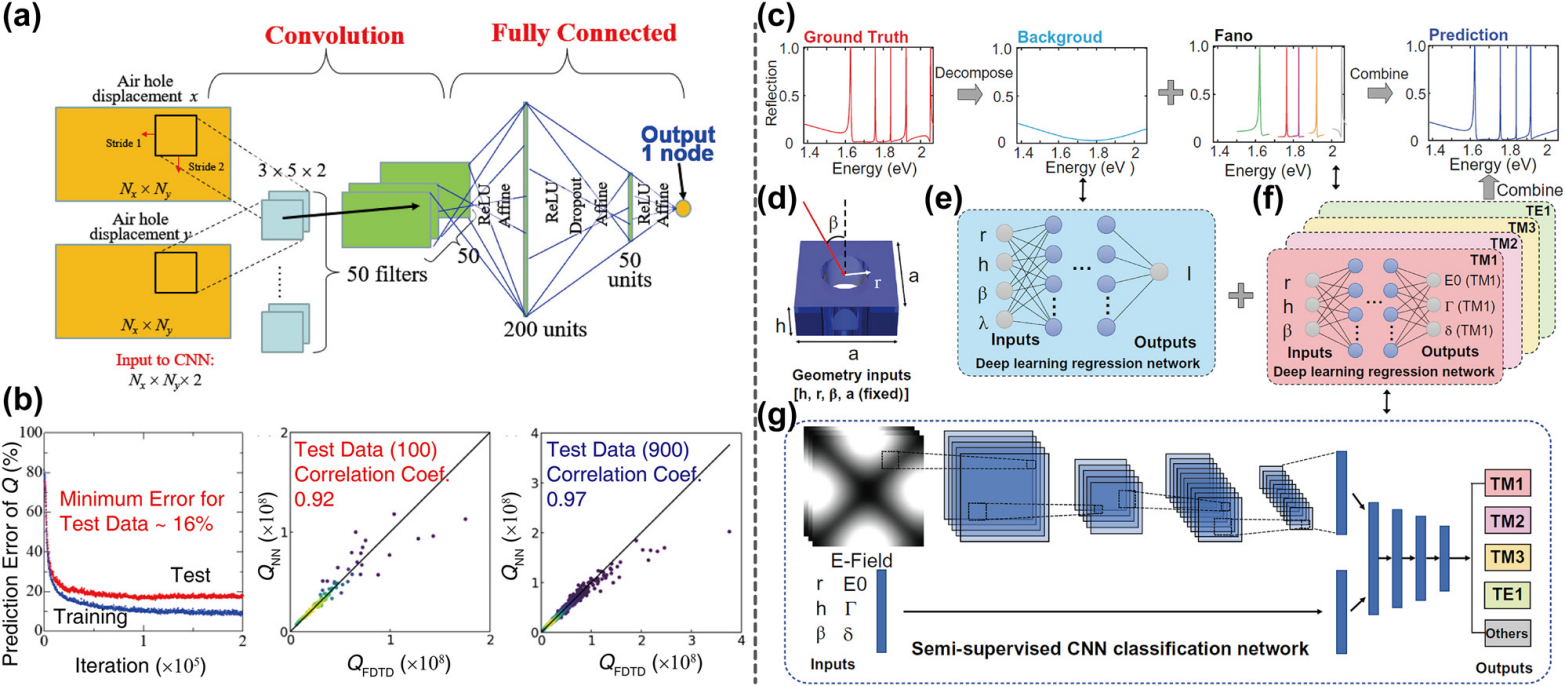
Inverse design for the quality factor. (a) The architecture of the neural network for quality factor prediction. (b) The results of the trained neural network. Left: the learning curve of the neural network. Middle: the correlation between the predicted and actual quality factors for the test data set. Right: the correlation between the predicted and actual quality factors for the training data set. (c) Schematic of the decomposition and reconstruction of a reflection spectrum. (d) The geometry inputs of the neural network. (e) The network for the background secra prediction. (f) Four networks for the Fano resonances prediction. (g) The architecture of the classification network. (a)–(b) Are adapted with permission from Ref. [67], and (c)–(g) are adapted with permission from Ref. [68].

CNN is a DNN that contains convolutional layers [26]. It is very powerful in learning spatial features of the input data, and is widely used in image processing. In 2018, Asano and Noda [67] constructed a CNN to learn the correlation between the structure of 2D photonic crystal nanocavities and their *Q* factors, and then applied it to inverse design high *Q* nanocavities. A heterostructure cavity was considered, where a line defect was introduced in a 2D photonic crystal. The displacement vectors of surrounding air holes were configured to be the input of the CNN, and it was expected that *Q* factors of input structures can be predicted accurately after operations through one convolutional layer and three fully connected layers as shown in Figure 4(a). The CNN was trained with a data set generated by the 3D finite difference time domain (FDTD) method, and converged successfully with a low prediction error and a good correlation coefficient between the neural network generated *Q* (*Q*_NN_) and FDTD generated *Q* (*Q*_FDTD_) (see Figure 4(b)). With a well-trained prediction model, optimizations were conducted by estimating the gradient by backpropagation and updating the structure accordingly. As the CNN greatly improved the computational speed of gradients, a 2D photonic crystal nanocavity with a *Q* factor as high as 1.58 × 10^9^ was obtained effectively after ∼10^6^ iterations. In comparison, this method outperformed genetic algorithms and intuition-based algorithms with fewer computational efforts and more tuning parameters. Nonetheless, there is still a problem that the performance of the CNN deteriorated in the high *Q* cases, as the randomly generated training data was rare in this region. This issue was resolved later with a proposed iterative optimization method [72]. The whole optimization process was divided into many rounds, in each round a standard neural network training was performed, and the trained neural networks were used to search other high *Q* structures using a similar gradient-based strategy. After the searching, High *Q* candidates were further determined through the first principles calculation, and were added into the training set ready for the next round of the optimization. This method can automatically accumulate high *Q* structures, thus improved the accuracy of neural networks in predicting their *Q* factors. An L3 cavity (2D photonic crystal cavities with three missing air holes) with a *Q* factor exceeding 1.1 × 10^9^ was designed within a total search of 8090 cavity structures using this algorithm.

While a large number of pre-calculated data are still required to train these neural networks, the deep reinforcement learning can alleviate this computational burden, as it doesn’t need any pre-calculated data and can effectively sample them during the exploration and the optimization. Li et al. [73] proposed a reinforcement learning algorithm called learning to design optical-resonators (L2DO) to optimize different kinds of photonic crystal nanocavities with high *Q* factors. The policy element of L2DO gradually learnt to take proper actions (modifications of the structure) according to current structures for more rewards (specified FoMs), thus L2DO can design high *Q* nanocavities step by step. The problem of needing large training sets can also be handled by utilizing the priori-existing physical laws to regularize the neural networks. Ma et al. [68] designed a resonance informed deep learning strategy to predict and inverse design high *Q* resonances for photonic crystal slabs. To accurately predict the spectrum of the structure, the task was divided into two parts, predicting a smooth varying background and predicting key parameters of resonances in the Fano equations, and they were used to reconstruct the final spectrum together as shown in Figure 4(c). Given the setup inputs, a deep learning regression network was used to give the background spectrum, and similar regression networks were used to predict resonances’ parameters for different bands (see Figure 4(d)–(f)). To obtain the training data, a decomposition algorithm was used to separate resonant peaks from background spectrum for simulated data, and a CNN was applied as a classifier which can efficiently recognize the bands that resonances belong to (see Figure 4(g)). Due to this regularization of the model using physical knowledge, a much smaller amount of data was needed to obtain a very accurate model. And with the help of its reliable and rapid predicting capabilities, it was employed to design a bound state in the continuum (BIC) with infinite *Q* factor with an interior point algorithm.

**Figure 5:**
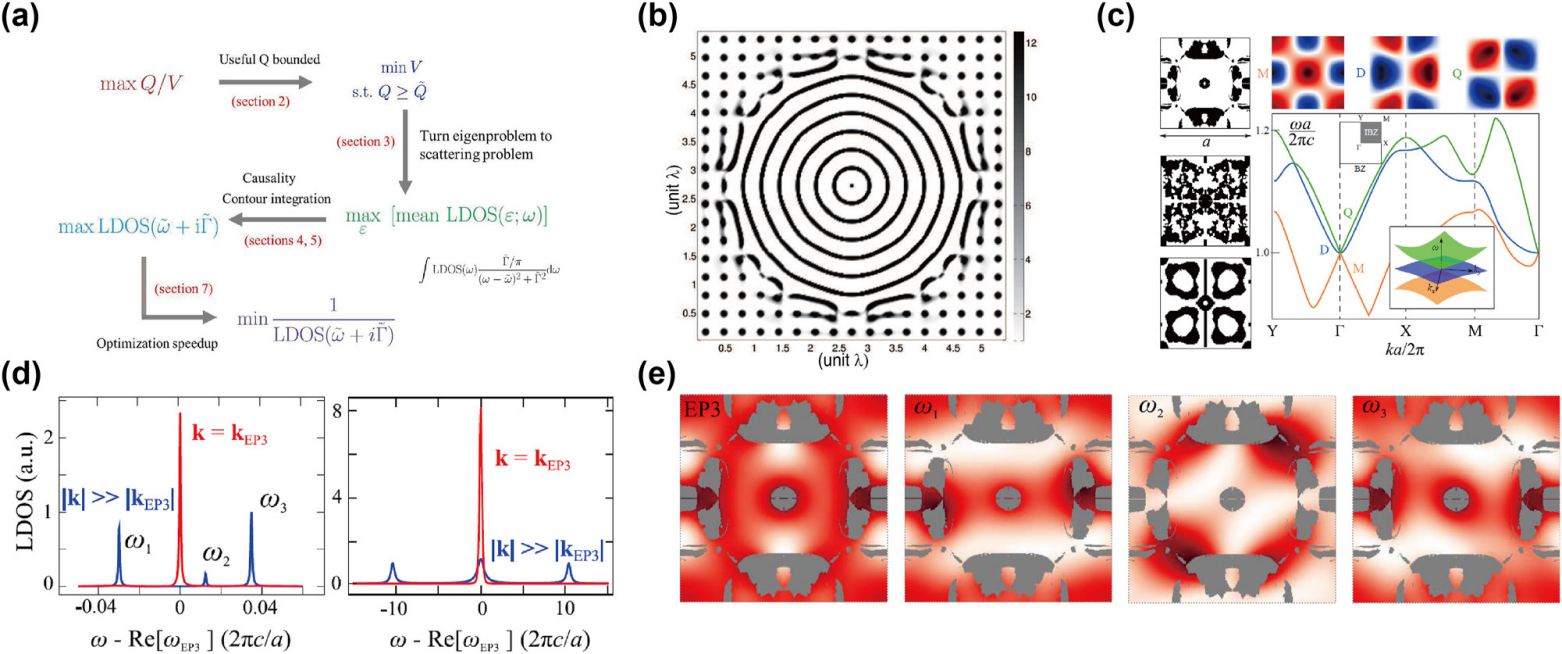
Inverse design for the local density of states. (a) Formulation of the optimization for the local density of states. (b) The optimized cavity from a photonic crystal initial guess. (c) The optimization results for third-order Dirac exceptional points. Left: the optimized structures for refractive indices = 2, 3, 1.82 (upper, middle, lower panels). Lower right: the optimized band structure with refractive index = 2. Upper right: the field distributions of different modes. (d) LDOS spectra at the center of the unit cell. (e) Spatial LDOS profiles evaluated at different frequencies. (a)–(b) Are adapted with permission from Ref. [75], and (c)–(e) are adapted with permission from Ref. [76].

Together with the *Q* factor, the modal volume *V* is always considered for resonances at the same time [77]. It gives more information about modes’ spatial distributions, and a smaller modal volume means a spatially more confined electromagnetic field. For the design of cavities, it is always desired to obtain high *Q* modes with small *V*, which can be expressed by the Purcell factor *Q*/*V*. Under the low-loss approximation, it is shown that LDOS is closely related with the Purcell factor by the equation $\text{LDOS}\approx \frac{2}{\pi \omega }\frac{Q}{V}$ [78], [79], and because the optimization of the LDOS is easier to implement, this helps to open the degrees of freedom for designing modes’ patterns. In 2013, Liang and Johnson [75] first formulated this optimization problem in a way shown in Figure 5(a). They noticed that a direct optimization of the Purcell factor (the ratio between the *Q* factor and the modal volume) is ill-posed as it can reach infinity, thus the problem was transformed to minimizing the modal volume for a given lower bound on *Q*. Then because the Purcell factor is actually an approximation to the LDOS, the LDOS was set as the FoM, which turned an original non-Hermitian eigenvalue problem to a scattering problem, and this can be solved effectively by the adjoint method. Under this formulation, the bound on the *Q* factor was achieved using a frequency-averaged LDOS with a window function, and the multifrequency calculation was circumvented by the contour integration, thus there was only one complex frequency scattering problem that needs to be solved. Finally, an inverse operation was performed to avoid slow convergence due to the sharp peaks in FoMs. Photonic crystal nanocavities with high *Q* factors and low mode volumes were successfully generated using this algorithm as shown in Figure 5(b). As the LDOS can be obtained by calculating the radiation power of the placed current dipoles, the resonance’s modal profile can be designed by properly setting the position and orientation of the dipoles. Lin et al. [76] designed photonic crystals with third-order Dirac exceptional points with this approach. They set up the excitation dipoles according to the requisite symmetries for monopolar (M), dipole (D) and quadrupolar (Q) modes, and optimized the LDOS of these three modes at the same frequency simultaneously by a max-min formulation. Applying a topology optimization, structures that support accidental third-order exceptional points involving M, D, and Q modes were produced for different refractive indices as shown in Figure 5(c). Then the LDOS at the geometrical centers and throughout the structures were plotted in Figure 5(d) and (e). It was demonstrated that the peak LDOS can have an 8-fold enhancement at the third-order exceptional point than those way from it, and this led to a strong modification to the spontaneous emission rate for emitters in the structures, which can be used for further applications. A similar method was also utilized to design quantum spin hall effect-based topological insulators [80], where the frequency modulation of D and Q modes was acquired to obtain trivial and nontrivial structures. Then these structures were used to construct boundaries that support topological edge states.

### 2.4 Inverse design for topological properties

As the photonic crystal is periodic in the real space, the reciprocal space and band structure can be defined in accordance with the Bloch’s theorem, and this provides a suitable platform for the study of topological photonics with various topological properties, such as polarization singularities [83], [84], [85], winding numbers [86], [87], [88], and Zak phases [89], [90], [91]. As the study of topological photonics in photonic crystals is receiving increasing attention, the application of inverse design in this region is also under broad investigation.

On the one hand, inverse design can be incorporated into the design process to indirectly optimize the topological properties. In 2018, Lin et al. [92] demonstrated the capability of topology optimization in finding dual-polarization Dirac cones (DPDCs). They judiciously optimized the LDOS and can produce DPDCs that are precursor to non-trivial topological states. Then Chen et al. [80] took a step further and designed photonic TIs with ultra-wide bandgaps. They followed the physics-inspired method in designing photonic TIs, but applied inverse design to obtain doubly degenerate Dirac cones and topological trivial/nontrivial photonic crystals. Similar methods have been used to produce high-performance photonic TIs based on different mechanisms (quantum spin hall effect, quantum valley hall effect) [93], [94], different crystalline symmetries [95], [96], and different orders [52], [97]. On the other hand, inverse design can also be used to directly optimize the topological properties [54], [81], [82], [98]. And because of the complex and discontinuous nature of topological properties, deep learning algorithms are frequently used in these researches.

**Figure 6:**
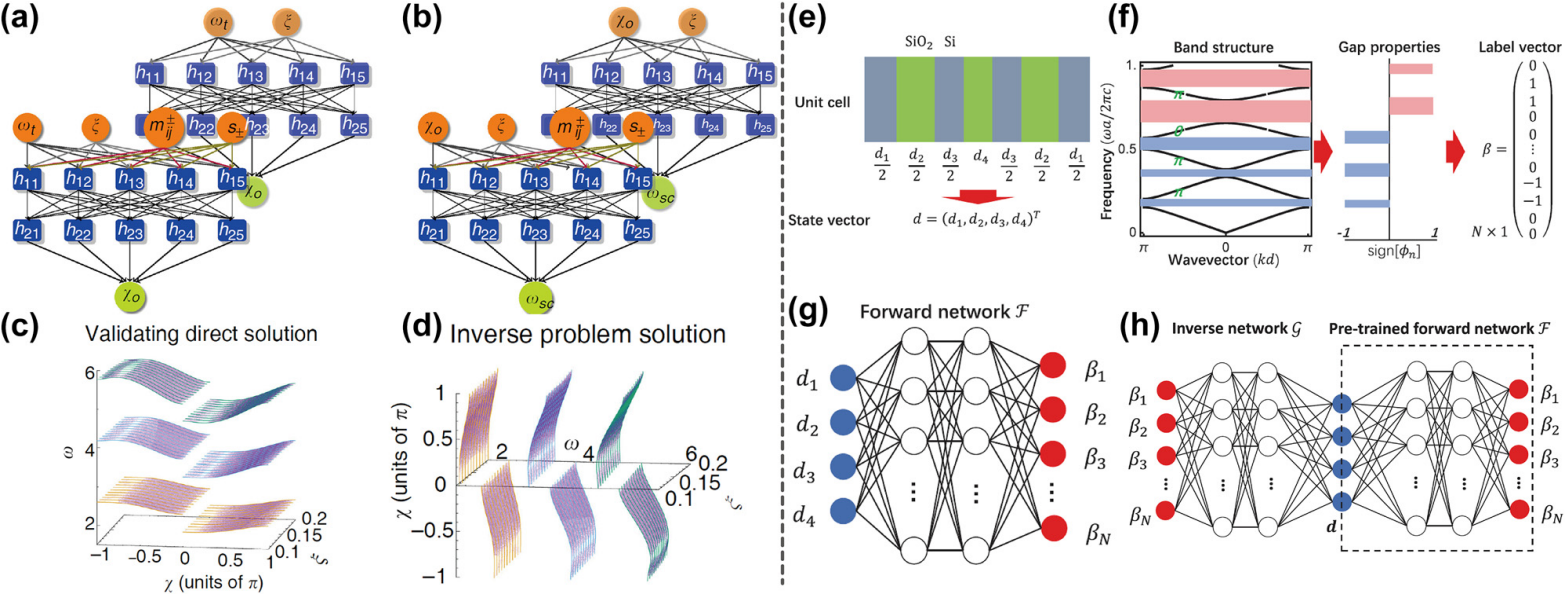
Inverse design for topological properties. (a) The architecture of the inverse design neural network. (b) The architecture of the forward prediction neural network. (c) The results of the forward prediction. (d) The results of the inverse design. (e) The state vector encoded from geometry parameters. (f) The label vector encoded from gap properties. (g) The architecture of the forward network. (h) The architecture of the tandem neural network. (a)–(d) Are adapted with permission from Ref. [81], and (e)–(h) are adapted with permission from Ref. [82].

In 2018, Pilozzi et al. [81] inverse designed topologically protected edge states at desired frequencies in the Aubry–Andre–Harper model (1D quasi-crystals that can be constructed from photonic crystals) with a regressive neural network. Regardless of the target frequency *ω*_*t*_ and a structure parameter *ξ*, some other categorical labels were also added as the inputs to solve the multivalued problem (see Figure 6(a)). The band gap label $m_{ij}^{\pm }$ was used to tackle the problem that the edge state frequency is a multi-mode function, which can have different frequencies for different modes under one geometry distribution. And the trend label *s*_±_ was introduced because one designated frequency may correspond to several structure parameters *χ*_0_ if the mode surface is not monotonic. And a similar neural network was constructed to predict the edge states’ frequency *ω*_*sc*_ for given structure parameters *χ*_0_ and *ξ*, as shown in Figure 6(b). For inverse design problems, some inputs may produce solutions unphysical because the target frequency can be out of the feasible range. Thus, a two-step self-consistent approach was proposed to verify the answer. In the first step, a desired *ω*_*t*_ was set as the input of the inverse neural network to produce the structure parameter *χ*_0_, and then the output was fed to the prediction neural network to generate the frequency *ω*_*sc*_. In the second step, the target frequency *ω*_*t*_ and the predicted frequency *ω*_*sc*_ were compared, and the design would be accepted only if their difference is small. Result of the prediction and inverse neural networks are plotted in Figure 6(c) and (d) respectively, which shows a good performance. Later, Long et al. [82] projected and designed 1D photonic crystals’ Zak phases with a tandem network consisting of a forward and an inverse neural network. They set the thickness of the 1D photonic crystals as state vectors d (see Figure 6(e)), and encoded the reflection phases’ signs of the band gaps into the label vectors (see Figure 6(f)). The Zak phases can be calculated from these signs through the equation

$$sign\left(\phi_{n-1}\right)/sign\left(\phi_{n}\right)={\text{e}}^{\text{i}\theta },\;\theta_{n}=0,\pi$$

where sign(*ϕ*_*n*_) is the sign of *n*th gap’s reflection phase, and *θ*_*n*_ is the *n*th band’s Zak phase. Setting the sates vectors as the input and the label vectors as the output, the forward model was a fully connect neural network with five hidden layers as shown in Figure 6(g). After the forward network had been trained, an inverse neural network was constructed in a similar fashion, but was trained in a tandem pipeline to resolve the one-to-many problem (see Figure 6(h)). During the optimization, the designed structures were evaluated by the pre-trained forward network effectively, then the differences between the target label vectors and the predicted label vectors were minimized to train the inverse neural network, and good results were obtained.

## 3 Inverse design for the response-based properties

Instead of the eigenproperties discussed above, the response-based properties, such as transmission, reflection and imaging under certain excitations are of more interest for the design of many devices. In these cases, the adjoint method is very suitable and often applied because of its formulation. We will focus on the optimizations on these characteristics for the photonic crystals in the following sections.

### 3.1 Inverse design for transmission

Transmission and reflection are very elementary properties for photonic devices ranging from photonic chips [99], [100], [101], [102] to virtual reality displays [103], [104]. On the photonic chips, many devices were used to control the flow of light based on its wavelengths, modes, and channels, such as wavelength demultiplexers, mode converters, hubs, etc., and they are all closely related to transmission and reflection. These devices are aligned together to implement complex functionality, and making them compact and highly efficient is an important optimization goal. The photonic crystals have contributed a lot to this field due to their ability to generate band gaps and confine the electromagnetic fields, and we concentrate on the optimization of transmission, which is the same for reflection.

**Figure 7:**
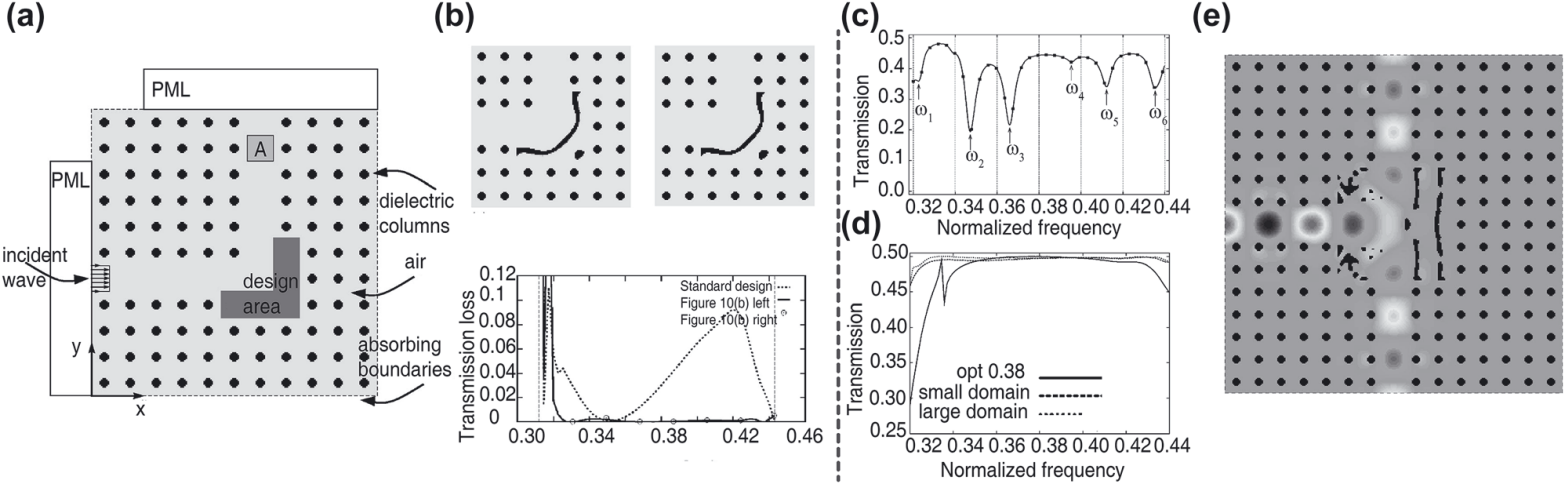
Inverse design for the transmission of on-chip devices. (a) Schematic of the optimization for 90° waveguide bends. (b) The optimization results. Upper left: the optimized corner structure. Upper right: the postprocessed corner design. Bottom: the transmission loss spectra of the optimized structures compared with a standard design. (c) Illustration of the active-set strategy. (d) The transmission spectra of the optimized structures. (e) The optimized structure with its field distribution. (a)–(b) Are adapted with permission from Ref. [101], and (c)–(e) are adapted with permission from Ref. [105].

In 2004, Jensen and Sigmund [101] designed low-loss waveguide bends that can deflect light by 90°. The problem set up is shown in Figure 7(a), the wave was incident from the left, and the upper region A was examined to calculate the transmission with the corner to be designed for higher efficiency, also perfectly matching layers were set to avoid unexpected reflection form the input and output ports. The adjoint method was used to effectively calculate the gradient with only one additional adjoint simulation, and the topology optimization was applied to optimize the corner geometry. They selected three frequencies to construct the FoM in order to enlarge the operation bandwidth, and obtained the structure shown in the upper left panel in Figure 7(b). Because this intermediate structure still contained pixels with values between 0 and 1, it was postprocessed to reach the fully discretized structure (see the upper right panel in Figure 7(b)), which is more practical for the fabrication. The results show that the designed structures possess loss lower than 0.3 % over a broad frequency range (*ω* ≈ 0.325 – 0.440(2*πc*/*a*)), compared with the standard design where only adding and deleting the dielectric columns are allowed (see the lower panel in Figure 7(b)). This indicates that devices with higher performances can be generated, when more degrees of freedom are available through inverse design methods. A similar approach was used to optimize photonic crystal waveguide Z-bends [106], and they applied min-max formulation for a large low loss bandwidth. The designed structure was fabricated utilizing e-beam lithography, and although there are some deviations between the fabricated structure and the designed one, its performance remained good enough with a nearly 1 dB low loss in the 200 nm wavelength range. This shows the robustness of the inverse-designed structures under the fabrication errors. Jensen and Sigmund [105] also proposed an active-set strategy with an artificial damping penalization to avoid unwanted local maxima induced by the resonances, and achieved a high-bandwidth low-loss T-junction waveguide. The active-set strategy separated the frequency range into *N* equally sized parts. After a regular number of iterations, a high-resolution transmission spectrum was computed with a fast-frequency-sweep technique benefitting from the Padé approximates, and the frequency with the lowest transmission was selected from each part to form a new FoM for later optimizations, as shown in Figure 7(c). Also, an artificial damping term was introduced to regularize the optimization landscape by smoothing out the effects of resonances, and the penalization of non-discrete design variables can be implemented similarly. During the optimization, the weight of the damping term was gradually reduced to turn the problem back to its original form. It appeared that when the design domain was confined to ten unit cells, a full transmission in the target frequency range (*ω* ≈ 0.32 – 0.44(2*πc*/*a*)) was unattainable, but the overall performance improved as the design domain increased (see Figure 7(d)), and the final structure with its field distribution at *ω* = 0.38(2*πc*/*a*) are shown in Figure 7(e). While many photonic-crystal-based devices such as mode converters, Y-splitters, and grating couplers were designed following alike routines [61], [107], other algorithms were also applied to optimize the structures [108]–[114]. Jiao et al. [110] applied the Wannier basis field expansion to formulate the relation between the input and output of the mode separators. Under this highly localized basis, adding and removing dielectric rods correspond to low-rank adjustments to the formulation, and a fast update can be obtained by taking account of this characteristic. With the ability to speed up the searching process, they used the simulated-annealing-based heuristic algorithm and successfully designed a compact separator for three guiding modes. Besides the optimization, it was demonstrated that a high fidelity and cost-efficient fabrication technique, called nanoimprint lithography, was able to produce topology-optimized structures with feature sizes under 30 nm, which is important for mass fabrication as the traditional electron beam lithography and deep-ultraviolet lithography are limited either because of the cost or the fabrication tolerance.

In addition to the on-chip devices, it is also important to optimize the transmission for free-space devices based on photonic crystals to achieve functionalities like focusing [117], diffraction [118], and analog optical computing [119]. There are works directly optimize the real space distribution of the transmitted light, for example, Hakansson et al. [120] used a genetic algorithm in conjunction with a fast and accurate simulation method based on multiple scattering theory to design flat lens with desired focal points. However, solving the optimization problems in terms of the momentum space is considered to be another enlightening perspective, as implied by the physical nature of photonics crystals.

**Figure 8:**
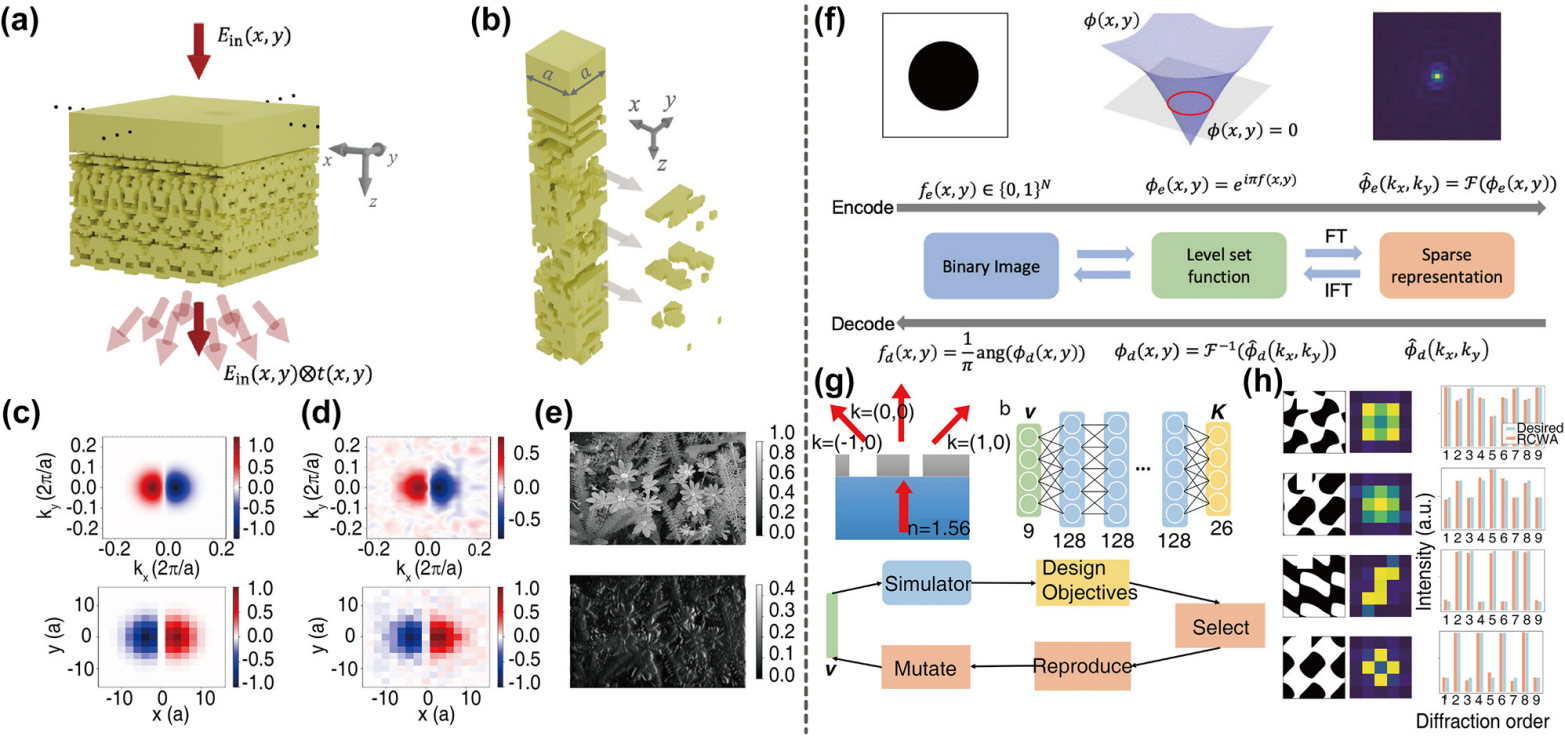
Inverse design for the transmission of free-space devices. (a) Illustration of the meta-crystals for general optical convolution. (b) The optimized structure of the meta-crystal for first-order differentiation. (c) The desired transmission function in the momentum space (top), and real space (bottom). (d) The optimized transmission function in the momentum space (top), and real space (bottom). (e) The simulation results. Top: the input image. Bottom: the output image. (f) Schematic of the Fourier transform-based encoding and decoding processes. (g) Illustration of the optimization strategy. Upper left: the cross section of the diffractive beam splitter. Upper right: the architecture of prediction neural network. Bottom: illustration of the modified evolution strategy. (h) The optimized diffraction patterns and the desired patterns. (a)–(e) Are adapted with permission from Ref. [115], and (f)–(h) are adapted with permission from Ref. [116].

In 2022, Wang et al. [115] designed meta-crystal slabs for general convolution operators, which are wide used in image processing and deep learning. According to the Fourier transform, convolution operations to the input electromagnetic fields in the real space correspond to multiplication operations in the momentum space with respective transmission coefficients. To enlarge the degrees of freedom for the optimizations, they chose “meta-crystals” whose periodicities were larger than the wavelengths, and tried to design its 3D structures to obtain desired performance zero order diffraction channel as shown in Figure 8(a). The distances between the ideal transmission coefficients and the current ones in the complex plane were set as the building unit for the FoM, and they were added with the designate weights and left with one phase degree of freedom to construct the final FoM. The optimized structure of a first-order differentiation kernel with a Gaussian envelope was shown in the Figure 8(b). The real part of its target transmission function in the momentum space, and the imaginary part of the associated real-space convolution kernel are shown in Figure 8(c), with the designed results in Figure 8(d). They also carried out a simulation, where the amplitude distribution of input electric field was a picture of flowers and leaves, and the output successfully highlighted their edges as expected by the kernel (see Figure 8(e)). Meta-crystal slabs supporting other convolution operations, such as second-order differentiation, have also been designed as required, and this shows the capability and flexibility of the algorithm. Liu et al. [116] proposed a Fourier transform-based encoding technique to assist the inverse design of diffractive optical elements. To make the binary structures compatible with frequency domain operations, they first transformed the binary images of the structures *f*_*e*_(*x*, *y*) into level set functions *ϕ*_*e*_(*x*, *y*), and then applied Fourier transform to the level set functions to encode the real-space information into the sparse momentum-space representations, while the decoding process was in a reverse order (see Figure 8(f)). With this topological encoding method, an eight-layer fully connected neural network was constructed to predict the transmission intensities of the nine diffraction channels for 3 × 3 diffractive beam splitters. Combined with this trained efficient simulator, they adopted a modified evolution strategy to globally search optimal structures that meet the required diffraction patterns (see Figure 8(g)). The encoded vectors *v* were generated randomly, and they were selected according to their performance to generate new populations after the reproduction and mutation. Several results are shown in Figure 8(h) with a good match between the desired patterns in blue and the designed patterns (evaluated by RCWA) in orange.

### 3.2 Inverse design for complicated properties

With the widespread application of photonic crystal-based devices, more complex properties are constructed from the basic properties to fully characterize the devices’ functionalities, and enable problem-specific as well as multi-objective inverse designs. Therefore, the optimizations for such properties are also important and are gaining more attention.

**Figure 9:**
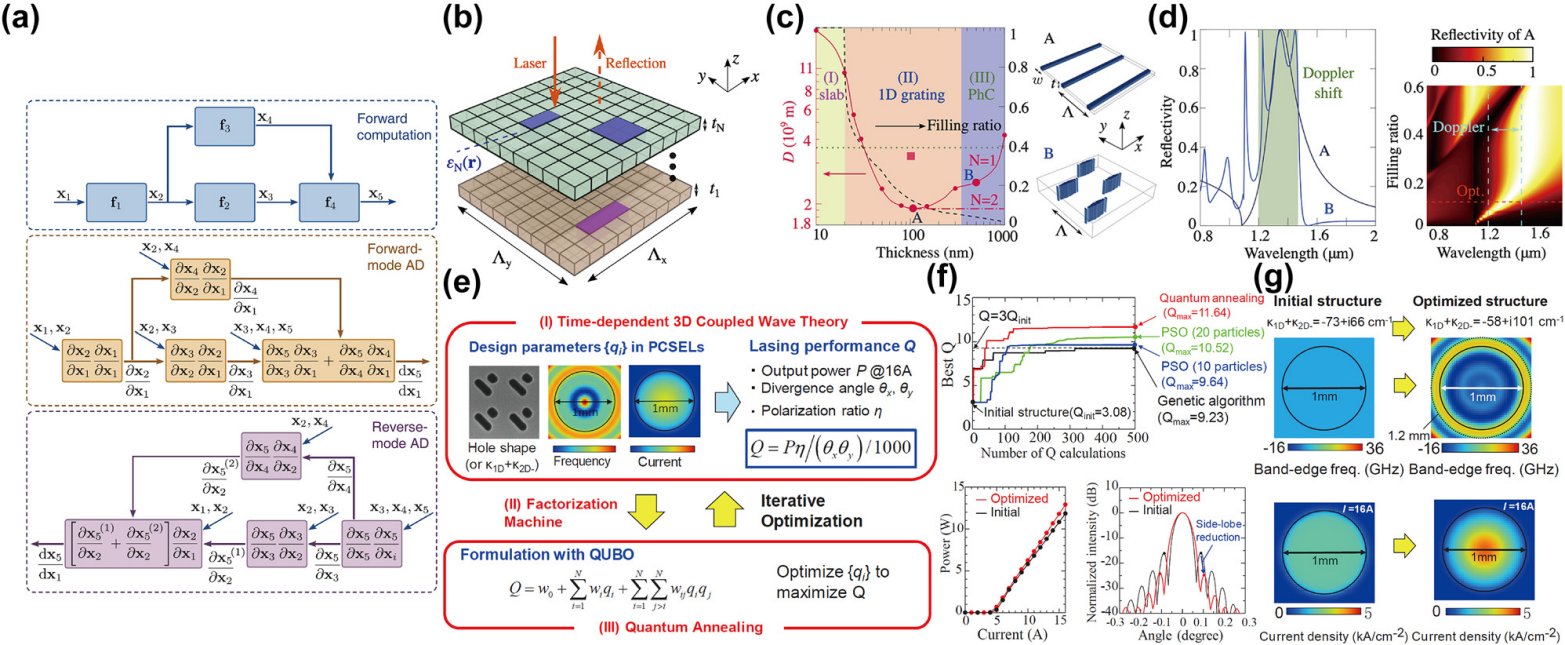
Inverse design for complicated properties. (a) Illustration of the automatic differentiation. Top: an example of the computational graph. Middle: the forward-mode automatic differentiation. Bottom: the reverse-mode automatic differentiation. (b) Schematic of the lightsail using stacked photonic crystal slabs. (c) The optimization results. Left: the optimized acceleration distance and filling ratio at different thickness for single-layer and double-layer structures. Right: examples of the optimized structures. (d) The optimized reflectivity spectra. Left: the reflectivity spectra of the two optimized structure in (c). Right: the reflectivity spectra of structure A at different filling ratios. (e) Schematic of the optimization procedure. (f) The optimization results. Top: the optimized *Q* for different algorithms. Lower left: the optimized output power. Lower right: the optimized far-field beam profile. (g) The optimized structure and the initial structure. (a) Is adapted with permission from Ref. [59], (b)–(d) are adapted with permission from Ref. [121], and (e)–(g) are adapted with permission from Ref. [122].

For gradient-based algorithms, the key point lies in obtaining the gradients with respect to the defined FoMs. In 2020, Minkov et al. [59] applied automatic differentiation, a concept from computer algebra and widely used in deep learning, to calculate the gradients for arbitrary output functions. During the calculation, the computational graph was constructed from generic functions with known gradients (see the upper panel in Figure 9(a)). It showed how the outputs were generated from the inputs through a series of operations. More importantly, according the chain rule of the differentiation, the gradients can be computed automatically by tracing the computational graph either from the inputs to the outputs, which is called the forward mode (see the middle panel in Figure 9(a)), or from the outputs to the inputs, which is called the reverse mode (see the lower panel in Figure 9(a)). Because for inverse design in photonic crystals there is always a large number of the input parameters (structural variables) and only one output (FoM), the reverse mode is computationally more efficient than the forward mode. This can be regarded as a generalization of the adjoint method, as the adjoint method is a reverse-mode case for the finite-difference or finite-element method. Utilizing this approach, Jin et al. [121] designed lightsails that use the optical force to accelerate to near the speed of light. They optimized stacked photonic crystals slabs and on the one hand tries to improve the broadband reflectivity, as this implies higher optical forces (see Figure 9(b)). On the other hand, lighter weights also contribute to the acceleration, thus a trade-off between these two quantities needs to be considered. They proposed a FoM representing the distance *D* that lightsails need to accelerate to target speeds, and used the method of moving asymptotes with gradients obtained from the automatic differentiation to optimize structures. Results for optimal single-layer and double-layer structures with different thicknesses and filling ratios are shown in Figure 9(c). It shows that three types of structures are favored at three thickness regimes indicated by yellow, orange, and blue, and two were plotted in the right panel. This phenomenon can be illustrated by the balance between the reflectivity and the lightsail mass for the overall performance at different thicknesses, and multilayer structures can further improve their FoMs. The reflectivity spectra are shown in Figure 9(d), while the left panel indicates different structures employ different mechanisms to achieve high reflectivities in broad bandwidths, the right panel shows that the reflectivity degrades as the filling ratio decreases, which is a typical trade-off as argued.

The application of non-gradient-based algorithms to complicated FoMs is relatively straightforward. Inoue et al. [122] used the quantum annealing to optimize photonic-crystal surface-emitting lasers (PCSELs), because it is well suited for solving a type of discrete optimization problem called quadratic unconstrained binary optimization (QUBO). As the output power *P*, divergence angles *θ*_*x*_ and *θ*_*y*_, and the polarization ratio *η* all needs consideration, they defined a dimensionless FoM *Q* based on above quantities to characterize the performance of PCSELs. Firstly, the design parameters were encoded into binary forms, and the time-dependent 3D coupled-wave theory (CWT) was used to analyze the lasing performance for 10 generated structures. Then, the coefficients of a FoM formulation based on QUBO were fitted with the analyzed results by applying the factorization machine. Finally, with this formulation they performed quantum annealing to find optimal structures that maximize *Q* (see Figure 9(e)). Results showed that the proposed quantum annealing method can reach higher *Q* values than other classical algorithms, and the designed structure possess higher output power, a smaller divergence angle and a larger linear polarization ratio than the initial structure (see Figure 9(f)). The comparison of the optimized structure and the initial one is shown in Figure 9(g) and a similarity exists between the inverse-designed band-edge frequency distribution and a previously proposed one. This suggests that the analysis of inverse design results can inspire new design insights.

## 4 Conclusion and outlook

In this review, we have discussed the recent development of inverse design in photonic crystals with various optimization strategies. From the bandwidth to the transmission and even for more complicated design targets, it is widely demonstrated that inverse design is a powerful tool that can always automatically and efficiently produce high-performance devices, sometimes outperforming previous designs. We further summarize the inverse design algorithms in Table 1 with their corresponding advantages, disadvantages, and potential tasks. We hope this will help researchers to choose proper inverse design methods, but the explorations also should not be limited by this general discussion. In addition, with the help of inverse design, it is possible to conduct systematic discoveries of the structures’ functionalities and characteristics. This helps to obtain the patterns and correlations between design parameters and objectives, which can lead to the research for some more fundamental questions, such as the performance’s physical limitation, and provide the guidance for future designs. Although much progress and achievement has been made, there are still many directions to be investigated in this rapidly growing field.

**Table 1:** Summary of optimization algorithms. Different optimization methods with their corresponding advantages, disadvantages, and potential tasks.

Algorithms	Advantages	Disadvantages	Potential tasks
Heuristic algorithm	– It is easy to implement	– It can be computationally intensive as	– Tasks that involve relatively simple
	– It has good global	for simulation time and the degrees of	geometries
	performance	freedom	
		– It needs fine-tuning and may suffer	
		from convergence issues	
Gradient-based algorithm	– It has good local	– It may have poor global performance for	– Tasks that have smooth
	performance	highly nonconvex optimization landscapes	optimization landscapes
	– It can deal with more degrees	– It can be computationally intensive	– Tasks that need a large number
	of freedom (for adjoint method	as for simulation time	of degrees of freedom (for adjoint
	and automatic differentiation)	– It mainly solves response-based	method and automatic differentiation)
		tasks (for adjoint method)	
Deep learning	– It is time-efficient after training	– It may require much computational	– Tasks that need a large number of
	– It has good global performance	resources during training	repetitive optimizations for some specific
		– It needs fine-tuning and may suffer	problems
		from convergence issues	
	– It can generate multiple	– Its trained models may lack generality	
	candidates (for generative models)	for different tasks	
	– It is good at recognizing		
	geometries’ patterns (for CNN)		

Firstly, more advanced optimization algorithms are needed to better search for globally optimal design structures. On the one hand, deep learning is stilling evolving very quickly with many exciting developments [123], [124], [125], thus it is important to incorporate them appropriately into inverse design of photonic crystals in order to obtain improved optimization results [126]. On the other hand, as the gradient of the FoM can be calculated in a general method, it remains to be explored how to use them wisely and regularize the problem for more efficient iterations. Moreover, hybrid algorithms combining both gradient-based algorithms and non-gradient-based algorithms worth investigation, as they can make use of the respective advantages of the two types of algorithms [127], [128], such as the former’s local convergence and the latter’s global exploration.

Secondly, while photonic crystals have rich physical properties, the inverse design with respect to some features is still relatively absent and should be further examined. For example, the polarization singularity in photonic crystal slabs is a concept of broad interest. Many questions, such as their evolutionary behavior [129], their association with the bound states in the continuum [130]–[134], and their link with the exciton-polaritons [135] are under extensive studies with potential applications. As the intuition-based algorithms are still dominant during the design process and limit the capability to freely control the polarization fields, it is desirable to apply inverse design techniques to facilitate the exploration in this field, and accelerate the transformation from scientific results into practical usage. Similar situations exist for topological properties. While there has been much work applying inverse design in this domain, the direct optimization of some topological invariants, such as the Chern number, topological charges, and exceptional points are not fully discovered yet and need further examination.

Thirdly, the optimization for multifunctional devices requires continuous development. As there has been a lot of works on optimizations for devices with a single target, it is natural to extend this to the multifunctional case, which is more challenging yet useful. It is difficult to deal with multiple objectives simultaneously with traditional methods, whereas inverse design can solve this problem to certain extent and has achieved some success, for example, multiple band gaps [48], dual-polarization topological insulators [50], and complex transmission patterns [115] in the momentum space. However, the performance inevitably deteriorates with increasing targets [59], and how to balance and maybe decouple them during the optimization, especially in the constraints of periodic boundary conditions, is an important problem that can help push inverse design a step forward in the field of photonic crystals.

Lastly, the optimization for multiphysics processes can extend the application of inverse design. Although many researches have focused on scenarios involving only optics, there are also plenty of demands for the optimization including multiple physical fields, such as the photoacoustic imaging [136], electro-optic modulations [137], and exciton–polariton interactions [138]. The coupling between different physical fields makes the problem more complex, and impedes the intuition-based methods. It is an attractive direction to apply inverse design in this region, and this will contribute a lot for the blending between the photonics and other disciplines.

In conclusion, the interplay between inverse design and photonic crystals has unveiled unprecedented opportunities in discovering fundamental physical principles and creating novel devices with superior performance and efficiency. With continued exploration in this interdisciplinary realm, there will be more innovative and significant advances, accelerating the evolution of the two fields and their fusion.
